# Characterization of *Lactobacillus*, *Bacillus* and *Saccharomyces* isolated from Iranian traditional dairy products for potential sources of starter cultures

**DOI:** 10.3934/microbiol.2017.4.815

**Published:** 2017-10-12

**Authors:** Farzad Rahmati

**Affiliations:** Department of Microbiology, Faculty of Science, Qom Branch, Islamic Azad University, Qom, IR Iran

**Keywords:** *Bacillus*, *Lactobacillus*, *Saccharomyces*, starter cultures, technological properties

## Abstract

The aim of the present study was to investigate technological properties of starter strains from traditional dairy products collected from five villages of Lorestan province in Iran. Thirty five samples were cultured on selective media (MRS broth, Nutrient Broth and YGC and then typical colonies checked for morphological features and eventually eighty two strains selected for further examination. The strains were evaluated for Hydrolysis of casein, starch and citrate, growth at 15 and 45 °C, growth in 4 and 6.5% NaCl, resistance to antibiotics (ampicillin, bacitracin, chloramphenicol, erythromycin, gentamicin, penicillin, novobiocin, nalidixic acid) proteolytic and lipolytic and acidification activities. Sixteen strains chosen according to the difference in cell morphology and were identified using API galleries and ability to metabolize various carbohydrates, which consequently, led to identifying seven *Lactobacillus casei*, five *Lactobacillus plantarum*, two *Saccharomyces cerevisiae* and two *Bacillus subtilis.* In general, two strains of *L. casei* AKL2, DDL2, two strains of *L. plantarum* SYL5, ACL4 and one strain of *S. cerevisiae* DDy2 was demonstrated the most important technological characterization that suitable for using as starter cultures.

## 1. Introduction

Since milk provides the conditions for microbial growth, such as water activity, pH close to neutral and reach of nutrients is a suitable place for many microorganisms. Procurement of all fermented dairy products depended on lactic acid bacteria (LAB) because of their ability to ferment lactose in milk to make predominantly lactic acid that creates a distinctive fresh flavor to the product [1]. Indeed, starter cultures containing selected microorganisms that add to milk or other products carefully under controlled and certain conditions to create desired changes. Starter culture microorganisms used in fermentations belong to family of bacteria collectively known as LAB. Raw milk and other fermented products due to technological reasons and to create a healthy profit to the consumer, *Lactobacillus* species added intentionally, or they are naturally present. Today, the Lab is an intensive focus of international research owing to their considerable role in fermented foods [2]. With population growth and the rising cultures of consumption of dairy products in Iran, dairy industries produce over thousands of dairy products. According to surveys, relevant industry in Iran totally depends on supply starter cultures from abroad. Nevertheless, prevalent application of dairy starter cultures, published research in Iran about dairy starter cultures are infrequent at the moment. Studies conducted in Iran in the field of *Lactobacillus* are more aimed at the food industry to optimize the quality of dairy products, biochemical characteristics and identification. In one study in order to investigate the effect of thermophilic and mesophilic bacteria in the production of dairy drinks research has been done [3]. The scope of this study was isolation and study of technological characteristic of starters *Lactobacillus*, *Bacillus* and *Saccharomyces* from traditional dairy products. By this effort not only, we are capable of isolating and preserve these strains for commercial applicability to prevent annual output of millions of dollars from the country, but also produce dairy products in accordance with Iranian taste.

## 2. Materials and Method

### 2.1. Sampling, preparing and enrichment

Since the Lorestan province is known for its abundant and various traditional dairy products, a total of thirty five samples containing cheese, whey, yogurt and yogurt drinks (Doogh) was collected from five villages near to Khoramabad city. The samples were maintained in sterile conditions as immediately cooled and eventually analyzed upon arrival to laboratory and primary pH of samples measured, aseptically.

In order to increase solubility of hard tissue of some samples, for homogenization, 5 g of each sample under sterilized condition were mixed with 45 ml sodium citrate 2% (w/v) by mortar for 1 min at 45 °C [4]. After 30 min, 10 ml of each prepared suspension was cultured separately in De Man, Rogosa and Sharpe agar (MRS) broth for *Lactobacillus* species anaerobically, Nutrient Broth for *Bacillus* species aerobically and YGC (Yeast Glucose Chloramphenicol) for Yeast species at 30 °C for 24 h, pour plate method, (all mediums from Quelab, Canada). Because of the growth of *Lactobacillus* in the oxygen-free environment, anaerobic conditions were achieved by jar and Gas-pack method (BBL Gas-pack anaerobic system S, VWR international haasrode, Belgium).

For heat treatment of *Bacillus* sp., they were heated at 80 °C for 15 min in a Bain Marie before plating (Memmert GmbH Co WNB 14, Germany). Samples were cultivated for 24 h, diluted by serial dilution and Then 1 ml of each dilution by pouring plate mixed separately with 15 ml of MRS agar, nutrient agar and YGC. Afterward, plates were incubated for 48–72 h according to already mentioned conditions. It should be noted that in order to distinguish acid producing colonies from others, bromocresol purple indicator was used in MRS agar. From the plates with countable colonies of bacteria and yeast, colonies with different morphological features were isolated. The Purity of the isolates checked twice by repeated streaking them into the proper agar plates [5]. The individual bacteria and yeast colonies were stored in 50% glycerol at −40 °C until complementary tests [6]. All strains were investigated by Gram staining, catalase, motility, nitrate reduction, hydrolysis of citrate, casein and starch, growth at 10 and 45 °C, growth in 4 and 6.5% NaCl and using a microscope.

### 2.2. Hydrolysis of casein, starch and nitrate reduction

Casein is a large protein that the white color of milk is derived from it. This examination was performed on milk agar which for procurement of it used from 50 g dry milk, 2.5 g yeast extract, 1 g glucose, 12.5 g agar, 5 g casein and 1 L distilled water. Strains were cultured on milk agar and plates incubated at 35 °C for 24–48 h. Existence a clear zone of around the bacteria due to exoenzyme of strain is a clue that strain could hydrolyze casein. Starch agar is a differential medium that assays the capacity of a microorganism to produce particular Exo-enzymes, including oligo-1, 6-glucosidase and α-amylase that hydrolyze starch. In order to prepare this medium, was used from 10 g potato starch, 3 g beef extract, 15 g agar, 5 g gelatin and 1 L distilled water [7]. Strains were cultivated on starch and incubated for 24 h at 37 °C and then checked after adding Iodine, which changes color from a yellow-brown to dark brown-black in the presence of starch, to the plates. In order to evaluate the ability to reduce nitrate by strains, was used for Nitrate broth containing 4 g peptone, 3 g beef Extract, 1 g Potassium and 1 g proteose peptone No. 3. After inoculating strains with medium, tubes were incubated for 24 h at 37 °C. Afterward by adding sulfanilic acid and α-naphthylamine to tubes, change color to red indicates the test is positive.

### 2.3. Fermentation of carbohydrates

The ability to ferment eight different carbohydrates, including fructose, galactose, glucose, lactose, maltose, melibiose, raffinose and Sucrose by strains in MRS broth and Nutrient broth, containing phenol red (0.5 g/L) was evaluated. Basic medium was prepared by eliminating of glucose, beef extract, and adding phenol red. 0.5% stock solution of each by a membrane filter 0.2 µm was prepared and sterilized. Afterward, 0.5 ml of them was combined with 4.5 ml of basic medium and incubated for 24 h at 37 °C. The results, based on the phenol red color changes to yellow were evaluated [8]. Furthermore the ability to making CO_2_ from glucose fermentation was investigated by inoculation strains in Durham tubes containing glucose and then incubating for 24 h at 37 °C.

### 2.4. Investigating antibiotic resistance

Resistance or sensitivity of the strains toward eight antibiotics was interpreted by using several antibiotic diffusion disks compared with MIC for strains, which have determined previously toward various antimicrobial agents. Therefore, at first 1% (approximately 10^7^ cells) overnight strains were added to MRS and nutrient broth with concentration 0.5 MacFarlane, and a certain volume of the mediums divided into plates, pour plate method. By solidifying mediums, antibiotic disks (Mast Co, UK) applied the surface. Antibiotics were used in following concentration: 10 µg ampicillin, 10 µg penicillin, 10 µg gentamicin, 30 µg chloramphenicol, 30 µg novobiocin, 30 µg erythromycin, 30 µg nalidixic acid, 130 µg bacitracin. The plates were incubated at 30 °C under aerobic condition for *Bacillus* sp. and anaerobic for *Lactobacillus* sp., after passing 24 h, inhibition zone measured. In general, strains were considered as resistance if the inhibition zone diameter was ≤10 for ampicillin, penicillin and gentamicin, ≤15 mm for chloramphenicol, novobiocin, erythromycin and nalidixic acid, ≤18 mm for bacitracin. Results were interpreted according to the cut-off levels published in CA-SFM (2011).

### 2.5. Evaluation of proteolytic activity

Proteolytic activity of yeast and bacteria was determined by casein hydrolysis on agar plates comprising MRS, YNB (Yeast Nitrogen Base) and nutrient agar mediums supplemented with 10% of skim milk poured and solidified. It should be noted that for the growth of yeast, 0.5% of casein, 2% of agar, 0.5% of glucose was added to YNB in pH 7.0 [9]. Afterwards preparing the mediums, 1% of isolates with concentration 0.5 MacFarlane that were fresh combined with basic mediums which pointed already. The plates were incubated anaerobically at 30 °C for *Lactobacillus*, aerobically at the same temperatures for *Bacillus* and yeast within 24 h. Strains with the protease enzyme which have grown on relevant mediums were characterized by their ability to produce clear zone after precipitation with 1 M HCl solution against an opaque background of unhydrolyzed protein and eventually, the clear zone diameter was measured.

### 2.6. Evaluation of lipolytic activity

Lipolysis can be described as enzyme-catalyzed hydrolytic cleavage of milk lipids, which resulting production of FFAs and partial glycerides [10]. These products, through accumulation of the proper concentrations and type of FFAs hydrolytically cleaved from milk fat cause desirable flavor. Lipase broadly applies in dairy products for hydrolysis of milk fat, cheese ripening and its fragrance and taste. In this test, lipolytic activity of strains that were isolated from cheese evaluated according to qualitative methods by Nile blue indicator. The strains were overnight incubated in the same condition previous test, cultivated on the medium by combining 0.5% (w/v) each of extracts yeast and meat, peptone, glucose, 1.5% of agar, 5% of the butter in Tween-80 1, 3, 5% [11]. After one-week incubation at 25 °C, colonies that their fat cells were blue or green-blue considered as lipolysis strains and lipolytic zone diameter measured.

### 2.7. Evaluation of acid production

This test relies on incubating overnight of strains in the skim milk medium. Strains were initially grown in MRS broth, Nutrient broth and YGC and then in sterile reconstituted skim milk supplemented with 0.2% of glucose and 0.3% of yeast extract for two sequential subcultures [12]. 1% of overnight activated cultures were inoculated with 100 ml of sterile reconstituted skim milk 10% and incubated for 24 h at 30 °C. pH shifts were ascertained using a pH meter (Teika Co, Japan) and strains considered as fast, medium, or slow acidifying when pH reached to below the isoelectric point, and milk began to curdle at intervals of time 6, 12, 24 h, respectively.

## 3. Results

In this research, eighty two strains using phenotypic methods were isolated from dairy products of Lorestan province and identified based on the carbohydrate fermentation test. In general, strains were gram-positive, catalase and motility negative, non-spore forming, and their cell features were bacilli, small chains of varying length or in a cluster considered as *Lactobacillus*. Since *Lactobacillus* strains were able to grow at 15 °C but did not grow at 45 °C, so they considered as mesophile *Lactobacillus* sp. [13]. Furthermore, due to lack of CO_2_ production from glucose known as heterofermentative *Lactobacillus* sp. Referring to the table fermentation of carbohydrates by mesophile hetrofermentative *Lactobacillus* in Bergey's manual, *L. plantarum* due to fermentation both raffinose and melibiose are distinguishable compared with *L. casei* was able to ferment neither of carbohydrates. Also, strains were gram-positive, nitrate reduction, catalase and motility-positive and had a shape of long bacilli chains, in clusters or pairs, containing oval or spherical spores in the center or terminal and were able to hydrolyze glucose, xylose, arabinose and manose, starch were known as *B. subtilis*. Moreover, large oval or spherical cells with a specific nucleus could able to hydrolyze glucose, sucrose, maltose, galactose, raffinose, But not able to use lactose and xylose identified as *S. cerevisiae*. The results of biochemical tests in Table 1 and carbohydrates fermentation and ability to produce CO_2_ from glucose are shown in Table 2, briefly.

### 3.1. Investigating antibiotic resistance

*Lactobacillus* sp. may act as a resource of antibiotic resistance gene that transfer within the gastrointestinal tract or the food chain to pathogenic bacteria [14]. It is necessary before using starter cultures to confirm that the bacterial strains involved do not include any genes for antibiotic resistance [15]. Indeed, strains with high resistance toward antibiotics are not appropriate for use as dairy starter cultures. Results of antibiogram test toward eight antibiotics are summarized in the Table 3. Generally, three bacterial strains SYL5 (*L. plantarum*), ACL4 (*L. plantarum*), AKL2 (*L. casei*) had the diameter less than the determined value for each antibiotic, so they were considered as resistant strains toward all antibiotics used in this test. This result is in agreement with that found by Sharma, who expressed *Lactobacillus* sp. isolated from probiotic have a low-level of resistance as an intrinsic characteristic toward ampicillin, chloramphenicol, gentamicin and penicillin and high level of resistance to nalidixic acid, vancomycin and streptomycin [16].

**Table 1.** Biochemical characteristics of strains.

Strain	Motility	Nitrate reduction	Hydrolysis of			Growth at temperatures		Growth on NaCl	

			Citrate	Casein	Starch	10 °C	45 °C	4%	6.5%
GYL1	−	+	+	+	+	+	−	+	−
DYL2	−	+	+	+	+	+	−	+	−
GYL3	−	+	+	+	+	+	−	+	−
BYL4	−	+	+	+	+	+	−	+	−
SYL5	−	−	+	+	+	+	−	+	−
SCL1	−	−	+	+	+	+	−	+	−
GCL2	−	−	+	+	+	+	−	+	−
DCL3	−	−	+	+	+	+	−	+	−
ACL4	−	−	+	+	+	+	−	+	−
AKL2	−	+	+	+	+	+	−	+	−
ADL1	−	+	+	+	+	+	−	+	−
DDL2	−	+	+	+	+	+	−	+	−
DDb1	+	+	+	−	+	+	−	+	−
SDb2	+	+	+	−	+	+	−	+	−
SKy1	n	+	n	+	−	+	−	+	−
DDy2	n	+	n	+	−	+	−	+	−

Legend +: positive; −: negative; n: no data. After 24 h incubation at an optimal temperature.

### 3.2. Investigating proteolytic activity

Production of high-quality fermented dairy products dependent on the starter bacteria proteolytic system [17]. Therefore, due to the significant role of proteolysis for improvement of flavor, the proteolytic ability of starter cultures is considered as an important phenotypic attribute. Protease activity leads to production of peptides and amino acids. Free amino acids and composed peptides have a direct effect on flavor or as precursors of the aroma-producing composition through secondary catabolic reactions perform a role in these products. Vuillemard et al. [18] argued if the lipolysis area diameter in the plates is between 15 and 21 mm, strain is called proreolytic. With regard to Table 4, strains displayed various protease activities, as six strains DYL2 (*L. casei*), SYL5 (*L. plantarum*), ACL4 (*L. plantarum*), AKL2 (*L. casei*), DDL2 (*L. casei*) and DDy2 (*S. cerevisiae*) exhibited relevant activity and had a diameter of between 15 and 19. This result is in agreement with findings of Ma et al. [19] that stated generally, *Lactobacillus* species isolated from milk products showed different proteolytic activity and strains with the highest lipolytic activity are able to lower pH significantly.

**Table 2.** Carbohydrates fermentation test and ability to produce CO_2_ from glucose.

Strain	Fermentation of									Production of CO_2_	Identification
	Fru	Gal	Glu	Lac	Mal	Mel	Raf	Suc	Xyl	Glucose	
GYL1	+	+	+	+	+	−	−	+	−	−	*L. casei*
DYL2	+	+	+	+	+	−	−	+	−	−	*L. casei*
GYL3	+	+	+	+	+	−	−	+	−	−	*L. casei*
BYL4	+	+	+	+	+	−	−	+	−	−	*L. casei*
SYL5	+	+	+	+	+	+	+	+	−	−	*L. plantarum*
SCL1	+	+	+	+	+	+	+	+	−	−	*L. plantarum*
GCL2	+	+	+	+	+	+	+	+	−	−	*L. plantarum*
DCL3	+	+	+	+	+	+	+	+	−	−	*L. plantarum*
ACL4	+	+	+	+	+	+	+	+	−	−	*L. plantarum*
AKL2	+	+	+	+	+	−	−	+	−	−	*L.casei*
ADL1	+	+	+	+	+	−	−	+	−	−	*L. casei*
DDL2	+	+	+	+	+	−	−	+	−	−	*L. casei*
DDb1	−	−	+	+	+	−	+	+	−	−	*B. subtilis*
SDb2	−	−	+	+	+	−	+	+	−	−	*B. subtilis*
SKy1	+	+	+	−	+	−	+	+	−	+	*S. cerevisiae*
DDy2	+	+	+	−	+	−	+	+	−	+	*S. cerevisiae*

Legend +: positive; −: negative; fru: fructose; gal: galactose; glu: glucose; lac: lactose; mal: maltose; mel: melibiose; raf: raffinose; suc: sucrose; xyl: xylose. After 24 h incubation at optimal temperature.

**Table 3.** Resistance and sensitivity toward different antibiotics.

Strain	Amp	Bac	Cam	Erm	Gen	Pcn	Nvb	Nx
GYL1	S	S	S	S	S	S	S	R
DYL2	S	S	S	S	S	S	S	R
GYL3	S	S	S	S	S	S	S	S
BYL4	S	S	S	R	S	S	S	S
SYL5	S	S	S	S	S	S	S	S
SCL1	S	S	S	S	S	S	S	S
GCL2	S	R	S	S	S	S	R	R
DCL3	S	S	S	S	S	S	S	S
ACL4	S	S	S	S	S	S	S	S
AKL2	S	S	S	S	S	S	S	S
ADL1	S	S	S	R	S	S	S	S
DDL2	S	S	S	S	S	S	R	S
DDb1	S	R	S	R	S	S	S	S
SDb2	S	R	S	S	S	S	R	S

Legend r: resistant; s: sensitive; amp: ampicillin; bac: bacitracin; cam: chloramphenicol; erm: erythromycin; gen: gentamicin; pcn: penicillin; nvb: novobiocin; nx: nalidixic acid.

### 3.3. Investigating lipolytic activity

Lipolysis of milk fat by bacterial lipase lead to rancidity as short-chain fatty acids like butyric is released. Usually in the dairy industry to reduce bacterial lipase through heat treatment, however, may even survive certain lipases by ultra-high temperature treatment. Surrounding the fat milk by globule's membrane also is the most effective way as can by capturing milk fat, protect them from lipase, therefore, this test was performed in the industry and has particular importance in dairy product quality. Lipolytic activity of LAB is weak, nevertheless, they make cheeses from raw milk due to their lipolysis [20]. The results were achieved during implementation of this test, summarized in Table 4.

**Table 4.** Proteolytic and lipolytic activities of strains.

Strain	Proteolytic zone diameter (mm)	Lipolytic zone diameter (mm)		
		1% tween-80	3% tween-80	5% tween-80
GYL1	13	10.0	10.8	11.2
DYL2	18	−	−	−
GYL3	15	−	−	−
BYL4	15	−	−	−
SYL5	19	−	−	−
SCL1	16	−	−	−
GCL2	15	−	−	−
DCL3	16	−	−	−
ACL4	18	9.0	11.0	10.2
AKL2	19	10.5	10.2	11.5
ADL1	15	−	−	−
DDL2	18	11.5	11.0	10.0
DDb1	15	−	−	−
SDb2	14		−	−
SKy1	18	−	−	−
DDy2	19	9.5	9.0	8.8

Legend −: No proteolytic and lipolytic activities.

Five strains GYL1 (*L. casei*), ACL4 (*L. plantarum*), AKL2 (*L. casei*), DDL2 (*L. casei*) and DDy2 (*S. cerevisiae*) were capable of hydrolyze the fats. Therefore, this finding confirms that *Lactobacillus* sp. particularly *L. plantarum* has a weak lipolytic activity of *Lactobacillus* in compared with other bacterial species and has revealed more esterase activity [21]. This finding is in agreement with research were conducted by Oterholm, which demonstrated *L. plantarum* had the highest esterase activity among nine LAB species, and they could extract two esterase enzyme from bacteria.

### 3.4. Investigating acidification activity

**Figure 1.**
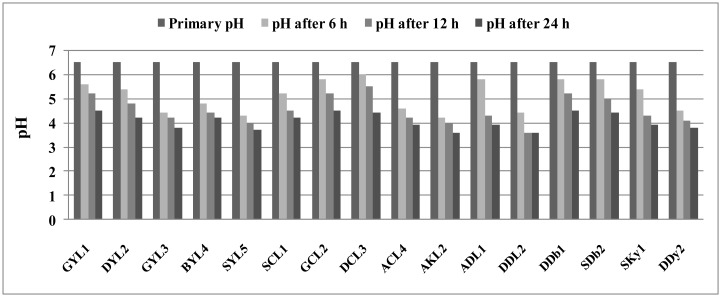
The pH changes at time intervals between 0 and 24 h in acidification test.

Lactic acid creates an acidic fresh flavor in dairy products and plays a significant role in curd tissue formation. *Lactobacillus* species are able to reduce pH through the acid production of sugars leading to, expansion of desirable sensory attributes, prevents the growth of pathogenic microbes to ensure durability and safety of final products [22]. In this study six strains within 6 h, four strains within 12 h and six strains within 24 h were able to reduce pH below the isoelectric point (less than pH = 4.6) and produce curd. Meanwhile, pH shifts of all strains were measured by pH meter at intervals of 6 and 24 h (Figure 1). Therefore, it can be stated that all strains have shown proper acid production within 24 h which indicates the ability to produce acid by these bacteria and use of them as a starter. These results are in agreement with findings of Mangia et al. [23] that argued *Lactobacillus* strains isolated from raw sheep's milk and Fiore Sardo cheese show ability to produce acid within 24 h as in this research five strains SYL5 (*L. plantarum*), ACL4 (*L. plantarum*), AKL2 (*L. casei*), DDL2 (*L. casei*) and DDy2 (*S. cerevisiae*) in during 6 h produced curd.

## 4. Conclusion

In earlier studies about *Lactobacillus* strains Vélez et al. [24] carried out a study on seven bio-yoghurts in Colombia that resulted in the identification Lactobacillus delbrueckii ssp. bulgaricus. Furthermore, the research conducted by Aplevicz et al. [25] demonstrates the presence of Lactobacillus paracasei and *S. cerevisiae* from the Brazilian sourdough. A similar research in Tunisia on raw camel milk to study the suitability of the strains for preparing of fermented dairy products, led to isolation hemofermentative and hetrofermentative *L. plantarum, Lactobacillus brevis* and *Lactobacillus pentosus* strains [14]. In sum, according to this research, we selected sixteen strains among eighty two strains according to the difference in cell morphology and then identified by API galleries and ability to metabolize various carbohydrates, which consequently, led to identification seven *L. casei* (GYL1, DYL2, GYL3, BYL4, AKL2, ADL1, DDL2), five *L. plantarum* (SYL5, SCL1, GCL2, DCL3, ACL4), two *B. subtilis* (DDb1, SDb2) and two *S. cerevisiae* (SKy1, DDy2). Eventually, based on various tests including Hydrolysis of casein, starch and citrate, growth at 15 and 45 °C, growth in 4 and 6.5% NaCl, resistance to antibiotics, proteolytic and lipolytic and acidification activities, five strains AKL2 (isolated from whey), DDy2, DDL2 (isolated from yoghourt drink), SYL5 (isolated from yoghourt), ACL4 (isolated from cheese), among sixteen strains from Iranian traditional dairy products presented the best performance for use as a starter or adjunct cultures in dairy industries. These strains had the highest sensitivity to antibiotics applied in this study and could able to hydrolyze casein and fatty acid in proteolytic and lypolitic test than other strains. Moreover, high acidifying activity in less time and lower pH scale makes them as a starter culture in producing dairy products. Summarily, these strains provide conditions for product procurement with traditional features on an industrial scale, but optimized use of them requires specific circumstance and further studies. Although it should be considered that molecular identity of strains should be accomplished to demonstrate the compatibility of these for commercial dairy products. Selected isolates in this study, which showed important intrinsic properties may supply extensive gene pool to develop genetically modified strains with unique specifications.
